# Changing Patterns in the Clinical Pathological Features of Hodgkin Lymphoma: A Report from Debrecen, Hungary

**DOI:** 10.5402/2011/810708

**Published:** 2011-12-01

**Authors:** Zsófia Miltényi, Zsófia Simon, Edit Páyer, László Váróczy, Lajos Gergely, Ádám Jóna, Árpád Illés

**Affiliations:** 3rd Department of Internal Medicine, Institute for Internal Medicine, Medical and Health Science Center, University of Debrecen, 4032 Debrecen, Hungary

## Abstract

*Introduction*. Hodgkin lymphoma shows a well-known geographic pattern, but temporal changes have been found recently as well. *Patients and Methods*. 439 Hodgkin lymphoma patients' clinicopathological and treatment data were processed in calendar periods of approximately ten years. The patients were treated at our department from 1980 until the end of 2008. *Results*. The first period (1980–89) contained 177 patients, the second (1990–99) 147, and the third (2000–08) 115 Hodgkin lymphoma patients. The mean age of the patients was 40.1, 35.9, and 36.8 years in order. The male/female ratio: 1.42, 1.45, 1.05 in order. Contrary-wise a unimodal age group pattern could have been seen with an incidence peak between 30 and 39 in the past decades. The incidence of classical mixed cellularity histological subtype is decreasing (43.7%, 58.23%, 42.6%, *P* = 0.0098 (it is only significant in the second period)); classical nodular sclerosis shows an increasing tendency (25%, 27.32%, 34.78%, *P* = 0.1734). The first incidence peak is predominantly created by classical nodular sclerosis, meanwhile the second peak by classical mixed cellularity. The number of early-stage patients (59.12%) is beyond the advanced stage (40%) in the last decade. Meanwhile, the number of second-stage patients was increasing (25.8%, 26.35%, 49.56%  *P* < 0.0001) and of patients in third stage was decreasing (53.4 %, 50.67%, 20%  *P* < 0.0001). The 5- and 10-year overall survival data were progressing: 59.7 %, 77.4 %, and 90.5 % and 44.1 %, 70.6 % and 90.5 % (expected survival) in the last decade. *Conclusions*. Changes can be explained by the altered nature of Hodgkin lymphoma, the changes in socioeconomic status and the development of diagnostic and therapy methods.

## 1. Introduction

Our knowledge about Hodgkin lymphoma (HL) is flaring apace. Nowadays it is obvious that the disease can be divided into two groups by its pathogenesis and clinical features: the nodular lymphocyte predominant (NLP) and the classic subtypes.

Due to the development of diagnostics and the prognosis-based risk-adopted treatment strategies, more than 90% of patients get into complete remission in the developed countries. The exact etiology of the disease is still unknown, however, is not uniform since genetic features, viral infections, and other factors such as different lifestyle habits can contribute to the development of the disease. The role of the Epstein-Barr virus is obvious. The so-called “two-disease hypothesis” [1], which proposes that the HL seen in the younger group is infectious in nature whereas that in the older persons has causes similar to other types of lymphomas. The incidence of HL shows differences worldwide, it is more frequent in developed countries, most common in North America, and less frequent in Asia [2]. Data from mid-1990s reported an increasing prevalence among young adults in developing countries and stagnant incidence in western countries [3]. In the USA from 1974 to 1996, the incidence of the disease was stagnant among white females, only decreased among white males; meanwhile, a definite increase of incidence can be found among black females. Until 1985 an increase after dropping can be found among black males [4]. As compared to the incidence of HL in the European Union in 2002 (males: 2.5/100000, females: 1.9/100000), in Hungary the disease is less frequent among males while is minimally more frequent among females (males: 2.1/100000, females: 2.1/100000). Among the twenty-five countries of the European Union (data not known in Romania and Bulgaria), Hungary is ranked the sixth place in the incidence of the disease among females and the twenty-second place among males [5]. In 2002 worldwide, the HL was the most frequent among males in Yemen, the less frequent in Egypt, and, additionally, less frequent than the average in Hungary [5].

Earlier epidemiological studies have revealed three patterns of the distribution in HL. Pattern 1, seen in developing countries and in patients of low socioeconomic status, shows an early childhood peak and a second peak around the age of fifty, with tumors predominantly of classical mixed cellularity (cMC) and classical lymphocyte depletion (cLD) subtypes with EBV positivity. Pattern 3, seen in developed countries and in patients of high socioeconomic status, shows a bimodal age group pattern with a peak incidence among young adults with tumors mainly of classical nodular sclerosis (cNS) subtype with EBV negativity. Pattern 2, seen in countries with transitional economies, has an early childhood peak and a second (female) decade peak with equal frequencies of the cMC and cNS subtypes [1, 2].

Considering the socioeconomic changes in Hungary, the authors regarded them as worthwhile to investigate, whether the pathological or clinical nature and the treat worth of HL have changed in the past decades.

## 2. Patients and Methods

The clinical data of 439 HL patients treated at the University of Debrecen, Medical and Health Science Center, Institute for Internal Medicine, 3rd Department of Medicine from 01/01/1980 until the 12/31/2008 were processed in a division of calendar periods of approximately ten years (first period: 1980–1989, second period: 1990–1999, third period: 2000–2008). 

Identification of histological subtypes was done according to Rye's classification [6], recently according to REAL/WHO classification [7]. Clinical staging was done according to the Ann Arbor Classification [8] and later with its Cotswold's modification [9]. The prognosis of the patients was given according to the EORTC recommendation [10] in early stage. In case of advanced-stage patients, prognosis was given according to the International Prognostic Index by Hasenclever and Diehl [11]. In advanced-stage disease, we considered the prognosis to be favourable, when the IPS value was 0–3, and unfavourable if it was at least 4.

The therapy of the patients occurred due to the current therapeutic protocols (radiotherapy, chemotherapy, or combined radio- and chemotherapy). Primary polychemotherapy was dominantly CV(O)PP (cyclophosphamide, vinblastine (vincristine), procarbazine, prednisone) until 1991, then COPP/ABV (cyclophosphamide, vincristine, procarbazine, prednisone/adriamycin, bleomycin, vinblastine) from 1991, recently ABVD (adriamycin, bleomycin, vinblastine, dacarbazine) from 1999. Salvage protocols were mainly BEACOPP (bleomycin, etoposide, adriamycin, cyclophosphamide, vincristine, procarbazine, prednisone), DHAP (dexamethasone, cytarabin, cisplatin), and CEP (CCNU, etoposide, chlorambucil, prednisone). The irradiation consisted of extended-field, mantle-field, converse Y, (sub)total nodal- or involved-field radiotherapy—initially with telecobalt machine, then from 2000 with linear accelerator. High-dose treatment and autolog hematopoietic stem cell transplantation were also accessible for treating relapsed or primary resistant patients from the 1990s [12]. 

Survival data were analysed using the Kaplan-Meier method by SPSS15 computer software. For the statistical analyses the chi^2^ test was used. *P* < 0.05 was considered significant.

## 3. Results

A total of 439 HL patients were treated primarily during the investigated period of 29 years. Male- to female-ratio decreased to 1.05 recently, instead of male predominance until now. The mean and median age was decreasing (Table 1).

**Figure 1:**
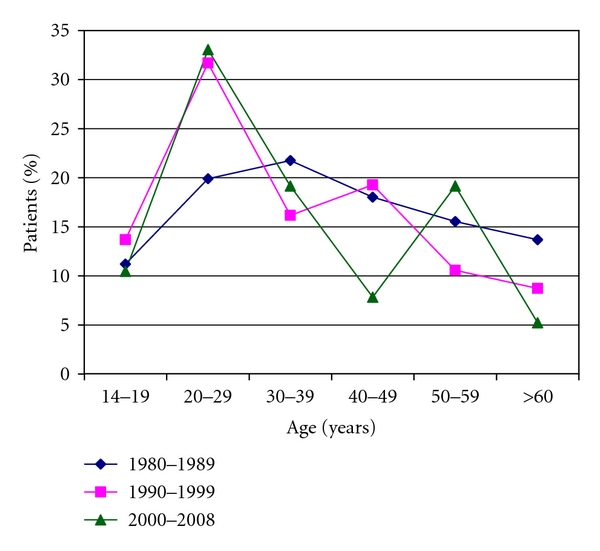
The age group pattern of Hodgkin's lymphoma patients during the investigated period.

Investigating the age of the patients, the age group pattern of the first period is unimodal, with one incidence peak between 30 and 39 years of age. We found a bimodal age group pattern in the second and third periods. The first incidence peak of this pattern can be found between 20 and 29 (like nowadays), the second peak between 40 and 49 in the second period, and between 50 and 59 in the third period (Figure 1).

**Figure 2:**
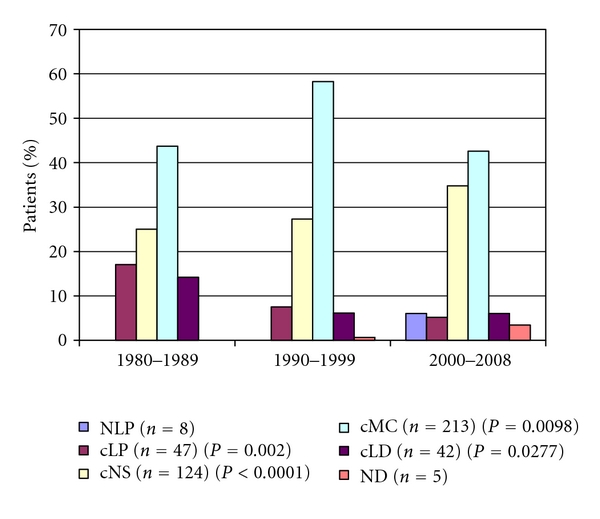
The pattern of the histological subtypes of Hodgkin's lymphoma patients during the investigated period.

The number of HL patients with classic mixed cellularity histological subtype was decreasing after a temporary increase (43.7%, 58.23%, 42.6%, *P* = 0.0098 (it is only significant in the second period)). The classic nodular sclerosis subtype shows a increasing tendency (25%, 27.32%, 34.78%, *P* = 0.1734). Classical lymphocyte predominant subtype (cLP) was decreasing (17.1%, 7.57%, 5.21%, *P* = 0.0038). The number of classical lymphocyte depletion (cLD) subtype was decreasing significantly also (14.2%, 6.19%, 6.06%, *P* = 0.0181), nodular lymphocyte predominant (NLP) histological subtype can be seen only in the past decade, when the REAL/WHO classification was used (Figure 2).

**Figure 3:**
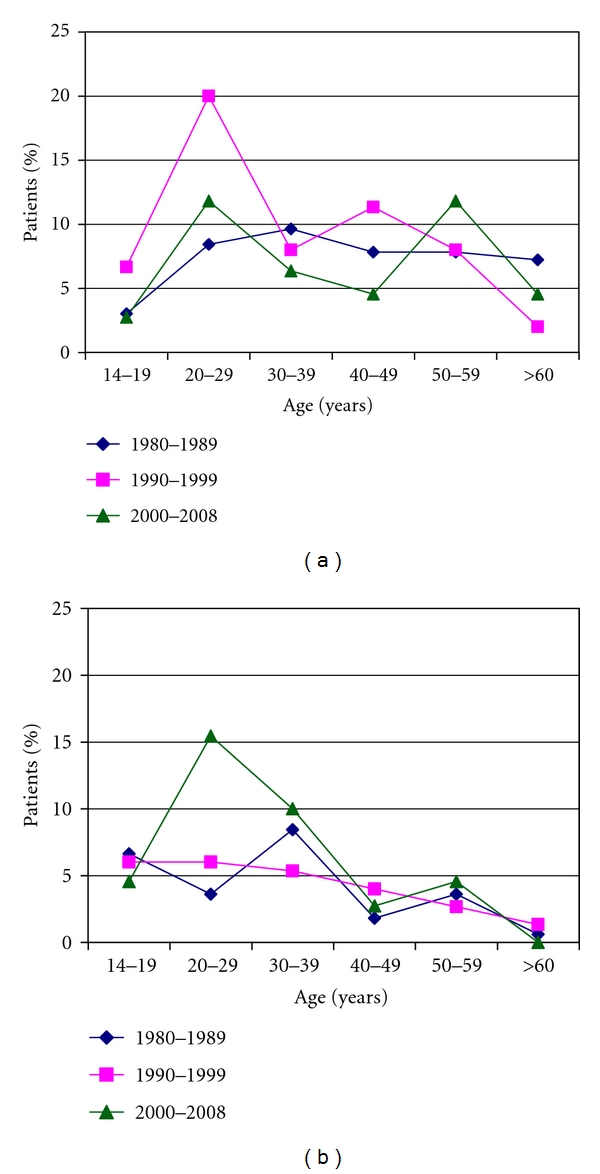
(a) The pattern of classical mixed cellularity histological subtype during the investigated period. (b) The pattern of classical nodular sclerosis histological subtype during the investigated period.

We found that the first incidence peak of the age group pattern is created predominantly by cNS; meanwhile, the second incidence peak is created by cMC histological subtype (Figures 3(a) and 3(b)).

**Figure 4:**
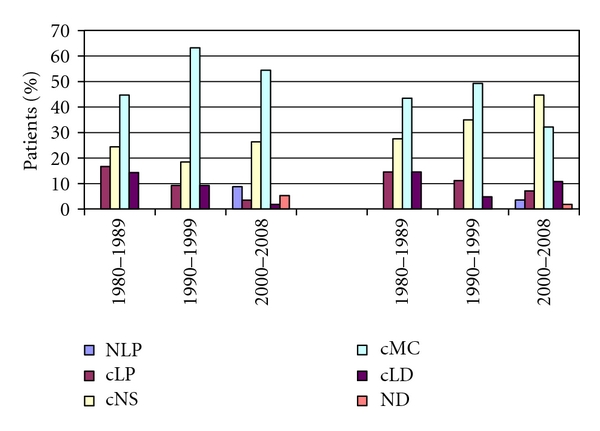
The pattern of the histological subtypes due to sexes during the investigated period.

Investigating the histological subtypes according to the sexual distribution, we found that cMC subtype is predominant among males in every period, its ratio is much lower among females, and in the third period cNS subtype became predominant. Among males the cMC subtype, meanwhile among females the cNS subtype, is the most common in the third period. The incidence of cLP and cLD subtypes decreased; cLD shows a slight increase in the third period again. Furthermore, NLP subtypes have been diagnosed in the last periods with an incidence of 6.08% (Figure 4).

**Figure 5:**
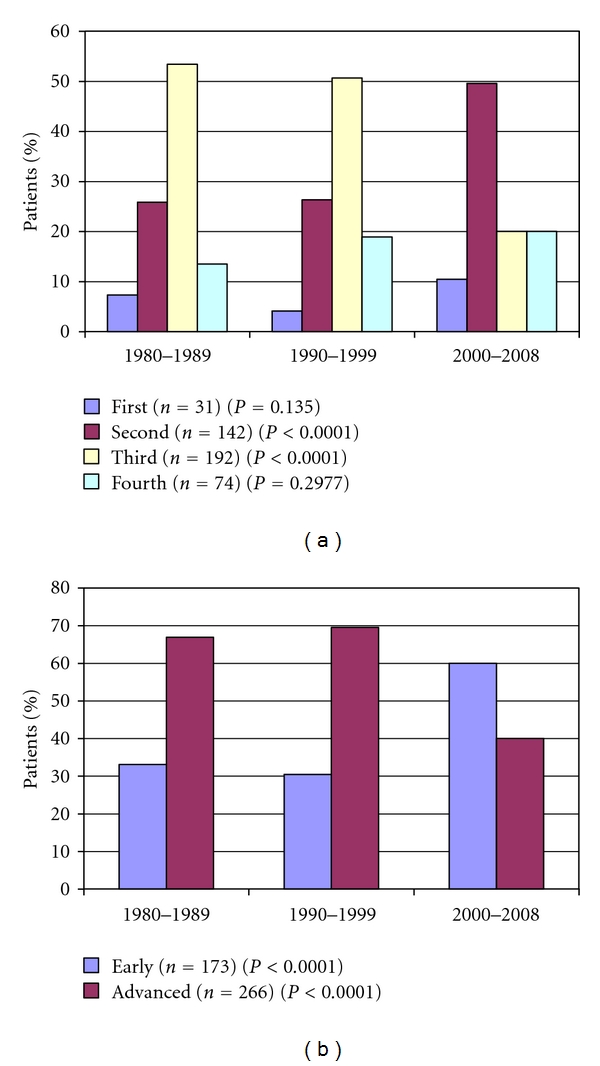
The distribution of the stages during the investigated period.

Comparing the early- (first-second) and advanced- (third-fourth) stage disease, we found an increased ratio of early-stage disease, and the difference was statistically significant. The increase comes on one hand from the significant growth of the number of second stage patients (25.8%, 26.35%, 49.56%, *P* < 0.0001) and on the other hand from the significant reduction of the number of third stage patients (53.4%, 50.67%, 20%, *P* < 0.0001). Nowadays half of the patients is diagnosed in the second stage of the disease (Figure 5).

From the first symptom (lymphadenomegaly and/or complain) until the diagnosis of the disease lasted an average 6.2 months in the first period. In the second period this only took 4.2 months and only 2.6 months in the third period.

We found that 45.59% of the early-stage patients and 28.26% of the advanced-stage patients were concerned to be in the unfavourable prognostic group.

The majority of the patients had B symptoms at the time of the diagnosis.

**Figure 6:**
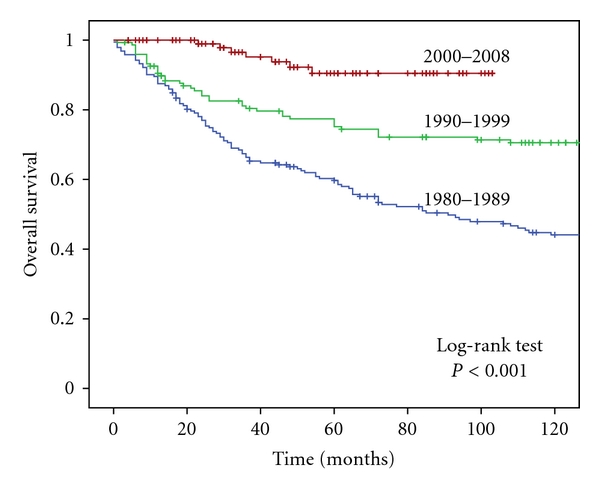
Overall survival during the investigated period.

The 5-year overall survival was 59.7%, 77.4%, and recently 90.5% (*P* < 0.001), and the 10-year overall survival data were also progressing: 44.1%, 70.6% and 90.5% (prognosed survival) in the last decade (Figure 6).

**Figure 7:**
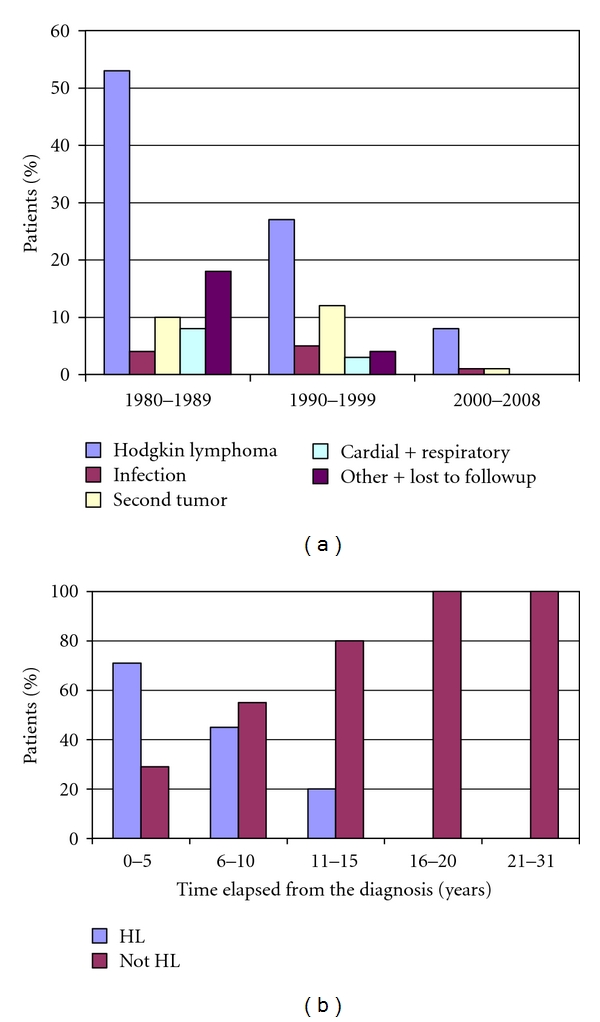
The distribution of the cause of death, HL: Hodgkin lymphoma, LFU: lost to followup.

During the investigated period, a total number of 154 patients passed away. More than 60% of them were diagnosed in the first period; they died predominantly of Hodgkin's lymphoma. The mortality coming from the basic disease was decreasing after this time (in the second period the basic disease mortality reduced into half), the remaining patients died of infection, second tumor, cardiopulmonary failure, and other factors (e.g., accidents) in nearly the same ratio. Comparing to the others, in the last period the mortality became minimal with still the predominance of the basic disease (Figure 7).

## 4. Conclusion

The incidence of HL was the greatest in North America (male: 3.2/100000, female: 2.4/100000), the smallest in East Asia (male: 0.3/100000, female: 0.1/100000), in Central and Eastern Europe ??? both males and females: 2.3/100000 in 2002 [2]. Data of Hungary from 2000 to 2003 reports an average incidence of 2.02/100000 among males and 1.59/100000 among females, but estimating Hungarian data, the fact must be considered that probably not every case was reported. Hence, the incidence of the disease can be considered average, both on the whole and due to sexual distribution [20]. Our current trial is not representative; hence, estimating incidence is not possible.

In Hungary 9 trials dealed with data of HL patients (Table 2). The duration of these trials differs significantly from each other (1 year [14] to 22 years [13, 19]); furthermore, these trials show great variability due to geographic differences (data from whole Hungary [14, 20] to investigate patients from only one county [13, 15]). Hence, exacting comparison and drawing conclusions is not possible, and attention can be attracted to further comprehensive trials.

Investigating sexual distribution generally male predominance was found; three trials found slight female predominance [12, 14, 15]. This ratio came from the female predominance in the cNS subtype in Kelényi et al. Estimating the results of Iványi et al. is hard, because among the diagnosed 86 patients only 66 patients' data were processed during their investigated period [15]. The mean age is around 40 in every trial (35.8–41.7 years) and shows a persistent slight decrease. The most common histological subtype—except for one trial—was cMC. Illés et al. presented countrywise data, whereas cNS was found to be the most common subtype during the investigated period of 4 years (between 2000 and 2003). Both uni- and bimodal age group patterns were found; the incidence peak in young adults around twenties was present everywhere. B symptoms were present unequivocally, more than half of the patients in every trial. The majority of the patients were diagnosed in the advanced stage, but it is hope worthy that both a trial in the current decade (2000–2003 countrywise data [20]) and our investigation's last period reports the diagnosis in early stage.

Between 1960 and 1997 in Scandinavian countries, in India, and in North America, the increase of the incidence of HL was found among teenagers and young adults. This increase was found rather among females and was primarily linked to the cNS subtype. In elderly rather a decreasing tendency was found, which was explained by the development of diagnostical methods [21]. A trial between 1984 and 1993 in the United Kingdom found the decrease of the incidence in both sexes, in every age groups, except for males between 1 and 24 [22]. Investigating seven European countries no changes was found in the incidence between 1985 and 1992 [23].

In our current study, the authors found the decrease of the number of patients, although the last period was one year shorter. The male/female ratio decreased as well, currently the number of male and female patients is almost equal. The mean age shows a decreasing tendency as well. Although the reason of this phenomenon is unknown, but shows similarity to the mentioned international data above [21].

Whereas we found a unimodal age group pattern in the first two periods, nowadays a bimodal age group pattern can be seen, with an incidence peak in young adults (20–29 years) and another in elderly (50–59 years), likewise to developed countries [2]. The fact that not all patients—especially the elderly—were treated in oncohematological centers can play a role in the explanation of the previously observed unimodal age group pattern. Hence, reporting the disease to the Hungarian Hodgkin's Disease Work Group did not occur every time. This phenomenon matches with the results of the last Hungarian survey [20]. 

Correspondingly, the distribution of the histological subtypes is characteristic to the developed countries. We found the significant decrease of the cMC subtype and the increase of the cNS subtype (not significantly). In the last period the incidence peak of young adults is created by the cNS subtype; meanwhile, the other incidence peak among elderly is created by the cMC subtype. Due to the introduction of the WHO's histological classification since 2000, the number of patients with cLP subtype decreased and with the new subtype, the NLP increased.

Patients diagnosed in the early stage become more frequent in the last period. The number of patients in the advanced stage decreased significantly, parallel with the significant increase of early-stage patients. Nowadays every second patient is diagnosed in stage two In the last decades the number of patients in stage three was decreasing continuously; accordingly, the number of stage two and early-stage patients were increasing continuously. This tendency suits with international authors' observation [24, 25]. Beyond the developing diagnostic methods (immunohistochemical examinations, developing imaging methods, like using PET/CT routinely), the patients' awareness (thanks to media, internet) and the highly educated workers in basic medical attendance can also play a role in the backstage of this phenomenon. Hence, the patients are treated earlier. Since, the patients are diagnosed in earlier stages, their chance to survive and their quality of life are continuously progressing, because of the lower dose and less toxic treatment at their case. Accordingly, the overall survive progressed.

According to international data, the 10-year overall survival of HL patients is 75–85% [20, 26], which are similar to our results. 5-year overall survival progressed, because of better treatment based on prognostic factors and better supportation.

The decrease of mortality—especially the mortality because of the basic disease—can be explained by developing treating methods as well. The mortality because of the basic disease fall to the half between the first and the second periods and fall to the third between second and third periods. Due to the more toxic polichemotherapy and extended-field, greater-dose irradiation used in the previous periods, the mortality coming from the late side effects of the treatment is still determinative. Only further follow can decide, whether how much can new treatment methods reduce these factors of mortality.

Investigating HL patients in the same geographical localisation in different periods gives an opportunity to observe the effects of different factors (e.g., viral infection, socioeconomic changes) on the characteristics of the disease [1]. Based on our results, it can be established that the clinical pathological features of Hodgkin lymphoma are changing in Hungary. We found the pattern to be characteristic to the developed countries out of the three epidemiologic patterns. Rising economy affects the socioeconomic status. The population is infected (e.g., EBV) fewer times. Beside the changes of these immunological features, much more environmental expositions affect people than previously stated. These facts and comprehensive (countrywise or different geographical locations) epidemiological trials can help in questing for the reasons, the treatment, and the survival of the disease.

## Figures and Tables

**Table 1:** The features of Hodgkin's lymphoma patients during the investigated period.

Period	1980–1989	1990–1999	2000–2008
Number of patients	177	147	115
Male	104 (58.75%)	87 (60%)	59 (51.30%)
Female	73 (41.25%)	60 (40%)	56 (48.70%)
Male/female	1.42	1.45	1.05
Mean age (years)	40.1	35.9	36.8
	(11–79)	(14–76)	(15–74)
Median (years)	38	33	32
B symptoms	92 (51.98%)	75 (51.00%)	59 (51.30%)

**Table 2:** Hungarian trials dealing with HL patients.

Author	Investigated period	Number of HL patients	Male/female ratio	Mean ages (years)	Most common histological subtype (%)	Age group pattern	B symptoms (%)	Early stages (%)	Advanced stages (%)
István and Giczy, 1979 [13]	1953–1974	166	1.81 : 1	Unknown	Unknown	Bimodal (15–40 and 50–65 years)	Unknown	Unknown	Unknown
Kelényi and Várbiró, 1980 [14]	1978	69	**0.97** **:** **1**	41.7	MC 45%	Bimodal (20–30 and 50–70 years)	Unknown	Unknown	Unknown
Iványi et al., 1988 [15]	1975–1986	66	**0.88** **:** **1**	35.8	MC 36.36%	Unknown	54.54%	34.80%	65.20%
Fleischmann, 1985 [16]	1978–1985	167	Unknown	Unknown	Unknown	Unknown	60%	16.90%	83.10%
Berényi et al., 1989 [17]	1983–1987	233	1.53 : 1	Unknown	MC 43.8%	Bimodal (10–20 and 40–60 years)	56.70%	41.60%	58.40%
Endrédi et al., 1991[18]	1987–1988	202	1.15 : 1	39.5	MC 42.6%	Unknown	Unknown	Unknown	Unknown
Illés et al., 1998 [19]	1975–1997	473	1.46 : 1	Unknown	MC 43.2%	Unimodal (20–30 years)	53.30%	33.30%	66.70%
Illés et al., 2004 [20]	2000–2003	611	1.27 : 1	39.6	**NS 44%**	Unimodal (20–30 years)	51.00%	**66.00%**	**34.00%**
Simon et al., 2007 [12]	1995–2004	163	**0.96** **:** **1**	36	MC 48.5%	Bimodal (20–30 and 50–60 years)	51.00%	41.10%	58.90%
Miltényi, Illés et al., 2008	1980–1989	177	1.42 : 1	40.1	MC 43.7%	Unimodal (30–40 years)	51.60%	33%	67%
Miltényi, Illés et al., 2008	1990–1999	147	1.45 : 1	35.9	MC 58.23%	Bimodal (20–30 and 40-50 years)	50.90%	31%	69%
Miltényi, Illés et al., 2008	2000–2008	115	1.04 : 1	36.8	MC 42.6%	Bimodal (20–30 and 50–60 years)	50.80%	**59.12%**	**40%**

## References

[1] Chang KC, Chen PCH, Jones D, Su IJ (2008). Changing patterns in the frequency of Hodgkin lymphoma subtypes and Epstein-Barr virus association in Taiwan. *Cancer Science*.

[2] Landgren O, Caporaso NE (2007). New aspects in descriptive, etiologic, and molecular epidemiology of Hodgkin’s lymphoma. *Hematology/Oncology Clinics of North America*.

[3] MacFarlane GJ, Evstifeeva T, Boyle P, Grufferman S (1995). International patterns in the occurrence of Hodgkin’s disease in children and young adult males. *International Journal of Cancer*.

[4] http://www.lymphomainfo.net/.

[5] http://info.cancerresearchuk.org/.

[6] Lukes RJ, Craver LF, Hall TC (1988). Report of the nomenclatura committee. *Cancer Research*.

[7] Harris NL, Jaffe ES, Diebold J (2000). The World Health Organization classification of neoplastic diseases of the haematopoietic and lymphoid tissues: report of the Clinical Advisory Committee Meeting, Airlie House, Virginia, November 1997. *Histopathology*.

[8] Carbone PP, Kaplan HS, Musshoff K (1971). Report of the committee on Hodgkin’s disease staging classification. *Cancer Research*.

[9] Lister TA, Crowther D, Sutcliffe SB (1989). Report of a committee convened to discuss the evaluation and staging of patients with Hodgkin’s disease: Cotswolds meeting. *Journal of Clinical Oncology*.

[10] Tubiana M, Henry-Amar M, Carde P (1989). Toward comprehensive management tailored to prognostic factors of patients with clinical stages I and II in Hodgkin’s disease. The EORTC Lymphoma Group controlled clinical trials: 1964–1987. *Blood*.

[11] Hasenclever D, Diehl V (1998). A prognostic score for advanced Hodgkin’s disease. *The New England Journal of Medicine*.

[12] Simon Z, Keresztes K, Miltényi Z (2007). [Our experiences in treating patients with Hodgkin disease in the last decade]. *Orvosi Hetilap*.

[13] István L, Giczy S (1979). Epidemiology of Hodgkin’s disease. *Orvosi Hetilap*.

[14] Kelényi G, Várbiró M (1980). [Malignant lymphoma reference center-1978]. *Orvosi Hetilap*.

[15] Iványi JL, Kiss A, Telek B, Pecze K, Rák K (1988). Experience with the treatment of Hodgkin’s disease (1975-1986). *Orvosi Hetilap*.

[16] Fleischmann T (1985). *Malignus Limfomás Betegeink Néhány Retrospektív Adata*.

[17] Berényi E, Szegedi G, Szabó K (1989). Comprehensive epidemiologic and clinico-pathologic study of Hodgkin’s disease. *Orvosi Hetilap*.

[18] Endrédi J, László T, Kelényi G (1991). Lymphoma referencia centrum 1987–88. *Orvosi Hetilap*.

[19] Illés Á, Vadász G, Gergely L, Szegedi G (1998). Hodgkin-kóros betegek kezelésével szerzett tapasztalataink. *Magyar Belorvosi Archivum*.

[20] Illés A, Keresztes K, Miltényi Z, Molnár Z (2004). A Hodgkin-kór hazai epidemiológiai és kezelési adatai. *Hematológia Transzfuziológia*.

[21] Cartwright RA, Watkins G (2004). Epidemiology of Hodgkin’s disease: a review. *Hematological Oncology*.

[22] Cartwright R, McNally R, Roman E, Simpson J, Thomas J (1998). Incidence and time trends in Hodgkin’s disease: from parts of the United Kingdom (1984–1993). *Leukemia and Lymphoma*.

[23] Cartwright R, Brincker H, Carli PM (1999). The rise in incidence of lymphomas in Europe 1985–1992. *European Journal of Cancer*.

[24] Glimelius B, Enblad G, Kälkner M (1996). Treatment of Hodgkin's disease: the Swedish National Care Programme experience. *Leukemia and Lymphoma*.

[25] Somers R (1999). *Treament Strategy in Hodgkin’s Disease*.

[26] Molnár Z (2004). A Hodgkin-kór kezelésének aktuális kérdései. *Hematológia Transzfuziológia*.

